# Quality of Life and Coping Strategies in Children with and Without Learning Disabilities from the Perspective of Their Parents and Caregivers

**DOI:** 10.3390/pediatric16040082

**Published:** 2024-11-07

**Authors:** Ayoob Lone, Abdul Sattar Khan, Fahad Abdullah Saeed AlWadani, Abdullah Almaqhawi

**Affiliations:** 1 Department of Clinical Neurosciences, College of Medicine, King Faisal University, Alhasa 31982, Saudi Arabia; mlone@kfu.edu.sa; 2 Department of Family Medicine, College of Medicine, King Faisal University, Alhasa 31982, Saudi Arabia; aalmughawi@kfu.edu.sa; 3 Department of Ophthalmology, College of Medicine, King Faisal University, Alhasa 31982, Saudi Arabia; falwadani@kfu.edu.sa

**Keywords:** learning disability, quality of life, coping strategies, children, Saudi Arabia

## Abstract

Background: Children with learning disability (LD) often experience a poor quality of life (QOL) compared to their peers without a known history of LD. Coping strategies are known to play a role in influencing their QOL. Objectives: This study aims to compare the QOL and coping strategies between children with and without LD. Additionally, it seeks to evaluate how coping strategies impact the QOL of children with LD in the Eastern Governorate of Saudi Arabia. Method: A representative sample of 6 to 18-year-old children with (n = 97) and without (n = 89) LD were recruited from different schools. The Short Form-12 (SF-12) health survey was used to assess both physical and mental health components, while the validated Coping Orientation to Problems Experienced Inventory (Brief-COPE) measured coping strategies. Data analysis included descriptive statistics (mean, standard deviation, percentage), independent t-tests, Spearman’s correlation, and binary logistic regression. Results: The results reveal that participants with LD show poor QOL in terms of role functioning, bodily pain, general health, vitality, social functioning, role emotion, and mental health in comparison to non-disabled children. Participants with LD show greater reliance on substance abuse and religious coping than non-disabled children. The results clearly indicate a fairly to moderately strong correlation between the physical component summary and all approaches to coping strategies except religious coping. Of all the approaches to coping methods, we observe a weak correlation among denial (*r* = −0.17, *p* < 0.05), substance abuse (*r* = −0.15, *p* < 0.05), and behavioral disengagement (*r* = −0.18, *p* < 0.05) with the mental component summary aspect of QOL. The results of logistic regression analysis indicate that grade (OR = 3.79; *p* = 0.01) is significantly related to LD. The physical component summary score is significantly associated with denial (*β* = −0.33, CI = −6.87–−2.19, *p* < 0.01), and substance abuse (β = −0.14, CI = −4.96–0.40, *p* < 0.05), while the mental component summary is significantly associated with active coping *(β* = −0.30, CI = −4.50–0.76, *p* < 0.01), behavioral disengagement (*β* = −0.20, CI = −4.48–0.30, *p* < 0.05), and humor coping strategy (*β* = 0.22, CI = 0.06–4.55, *p* < 0.05). Conclusion: These findings are relevant to researchers, psychologists, special educators, teachers, and clinicians, given the need to understand the coping variables to improve the QOL of these learning-disabled children.

## 1. Introduction

Learning disabilities (LD) represent a significant challenge for children worldwide, affecting their academic achievements, social interactions, and overall quality of life [1,2]. LD refers to several disorders that may affect the acquisition, organization, retention, comprehension, or the application of verbal and nonverbal information [3]. While extensive research has been conducted globally on LD and its impact on the quality of life (QOL) of children, there remains a gap in understanding this association within specific cultural and regional contexts, such as Eastern Province AlHasa, Saudi Arabia. Recognizing this gap, this study seeks to investigate the connection between learning disabilities, QOL, and coping strategies of children in this particular region. The prevalence of learning disabilities among children in Saudi Arabia is a matter of growing concern. Studies indicate that the prevalence rate ranges from 5% to 15% among school-aged children [4]. However, there is limited research exploring the impact of LD on the QOL of children in specific regions of Saudi Arabia.

Quality of life encompasses various dimensions, including physical, emotional, social, and academic well-being. Children with learning disabilities often face challenges in these domains, leading to decreased overall QOL [5]. Understanding the specific factors influencing the QOL of children with LD is crucial for developing effective interventions and support systems. Cultural and regional factors may significantly affect the manifestation and perception of learning disabilities and their consequences on children’s QOL [6]. Thus, investigating these factors within the context of Eastern Province AlHasa is essential for developing culturally sensitive interventions tailored to the needs of this population.

Numerous studies on coping strategies in relation to various illnesses and disabilities are available [7,8,9,10]. Recently, there has been increased focus on the topic of coping strategies in the context of learning disability [10,11,12,13,14,15]. Coping strategies are specific efforts, both behavioral and psychological, that a person uses to tolerate, reduce, or minimize stressful events [16]. The importance of coping strategies in reducing the negative impacts of disability on psychological and emotional well-being has been emphasized by several researchers [17,18]. Previous findings indicated that there are several coping strategies among people with learning disabilities for their psychological and emotional stress, which include proactive coping strategy [19], reactive coping [20], non-productive coping strategies (e.g., ignoring difficulties, not coping, and self-blame) [21], cognitive avoidance [22].

It is important to understand the coping mechanisms used by children with LD in order to develop targeted interventions and support systems that are tailored to their needs. Therefore, the primary aim of this study is to explore the association between QOL and coping strategies among children with learning disability in Eastern Province AlHasa, Saudi Arabia. By employing a cross-sectional design, the study intends to compare the QOL and coping strategies of children with LD to those of their neurotypical peers, identifying potential disparities and factors contributing to these differences. This research seeks to address a significant gap in the literature by examining the relationship between QOL and coping strategies in children with learning disability in Eastern Province AlHasa, Saudi Arabia. By elucidating this association within a specific cultural and regional context, the findings of this study aim to inform the development of targeted interventions and support services for children with LD in the region.

## 2. Materials and Methods

### 2.1. Study Design

This cross-sectional comparative group study examined the relationship between the QOL and coping strategies of children with and without LD between December 2023 and July 2024. This study received ethical approval from the Deanship of Scientific Research at King Faisal University in AlHasa, Saudi Arabia (KFU-REC-2023-SEP-ETHICS1350). The research was carried out in accordance with the principles outlined in the Declaration of Helsinki for research involving human participants. Prior to participation, all individuals were fully informed about the study’s objectives, and the survey was conducted only after fulfilling all ethical requirements.

### 2.2. Participants

The participants of the present study included 97 children aged 6–18 years diagnosed with LD. These participants were recruited from special education schools. Healthcare providers confirmed the diagnosis of learning disability in all participants by using the fifth edition of the *Diagnostic and Statistical Manual of Mental Disorders (DSM-5).* The inclusion criteria were established by reviewing the children’s developmental, medical, familial, and educational records and providing verbal informal consent from the children and their parents. Exclusion criteria included organic and functional disorders, children under the age of 6, those over the age of 18, and children with intellectual disability. The children without disabilities (n = 89) also satisfied the inclusion and exclusion criteria. We matched children with and without disabilities based on key demographic factors such as age, grade, areas of residence, and ethnicity.

### 2.3. Sample Method

Presently, no researchers have examined the QOL and coping strategies of children with LD in Saudi Arabia, and no previous study exists that allowed us to determine the sample size for this study. Although this was a cross-sectional study, we selected a convenience sampling method because we focused on a sample that is easy to assess and readily available [23]. Thus, we included 97 children aged 6–18 years diagnosed with LD and 89 normative children who met the inclusion and exclusion criteria. In addition to the ready availability of the sample, convenience sampling is also beneficial to researchers because the use of the method requires less time, money, and personnel than other sampling methods [24].

### 2.4. Data Collection Tools

We have used two questionnaires for data collection. These questionnaires are completed by parents or guardians on behalf of the children, with the children present during the process. While the SF-12 and Brief COPE are standardly used as self-report instruments, some studies employed parent proxy reports [25,26,27], especially in those targeting children with developmental or cognitive impairments. Although some bias may be present in parent-reported measures, using parent reports is typical for pediatric research when children are too young or have cognitive impairments that prohibit reliable completion of self-report assessments.

Short-Form-12: The quality of life was assessed using the SF-12 health survey, an abbreviated version of the SF-36 [28]. The SF-12v2 was shown to account for over 90% of the physical and mental summary scores derived from the SF-36 [29]. This self-reported tool evaluates eight health domains: physical functioning, physical role, pain, general health, vitality, social functioning, emotional role, and mental health. In this study, the physical component scale (PCS-12) and the mental component scale (MCS-12) each consisted of six items. These were calculated and standardized according to established guidelines [30]. Scores on the SF-12v2 range from 0 to 100, with higher scores indicating better health. A score of 50 or below on the PCS-12 suggests potential physical health concerns, while a score of 42 or below on the MCS-12 may indicate clinical depression [30]. The internal consistency of the physical and mental summary scales was robust, with alpha coefficients of 0.89 and 0.76, respectively [31]. In the present study, the Cronbach’s alpha values were 0.80 and 0.76, respectively.

Brief COPE: The coping strategies of participants were assessed using the Brief COPE inventory, a 28-item measure designed to evaluate both effective and ineffective coping responses to stress [32]. The Brief COPE includes 14 subscales that cover a range of strategies, such as self-distraction, active coping, denial, substance use, emotional support, instrumental support, behavioral disengagement, venting, positive reframing, planning, humor, acceptance, religion, and self-blame. Participants rated their use of each strategy on a 4-point Likert scale, from 1 (“not at all”) to 4 (“a great deal”). Scores for each subscale were obtained by summing the relevant items, with higher scores indicating greater use of the corresponding coping strategy [33]. Previous research has reported internal consistency reliability for the COPE inventory ranging from 0.42 to 0.89 [34]. In this study, the Cronbach’s alpha values for the Brief COPE ranged from 0.43 to 0.85.

Demographic and Clinical Variables: Demographic information, such as age, sex, living area, family type, income, occupation, housing status, and socioeconomic status, as well as clinical variables, including severity, comorbid conditions, and developmental history, were collected through parent/caregiver and medical records.

### 2.5. Procedure

Confidentiality was upheld, and cultural norms were respected during in-person interviews with the parents or guardians of the chosen children, which were conducted by senior medical students with training. Access to pertinent medical records and academic data was made possible by the cooperation between healthcare facilities and educational institutions. The parents or guardians gave their informed consent prior to the start of data collection, emphasizing that participation was completely voluntary. Strict procedures were adhered to in order to protect the privacy of the participants, making sure that all data were anonymized and safely stored. Before the data was collected, the questionnaire was translated and validated by a three-step process. Initially, two bilingual professors fluent in both English and Arabic translated the questionnaire into Arabic, and then the Arabic version was translated back into English by two other bilingual professors. In the second step, expert feedback and suggestions were integrated into the final version of the questionnaire In the third step, the finalized Arabic version was tested by 25 healthy volunteers from the local area as part of a pilot study to confirm the questionnaire’s reliability and validity. After this assessment, the specialists endorsed the final version, which was then administered through personal contacts.

### 2.6. Statistical Analysis

Statistical Package for Social Sciences (SPSS) software (version 27.0) was used for statistical analyses. Descriptive statistics were used to characterize the study population. The chi-square test was used to analyze categorical variables. The means of the children with learning disability and the normal group were compared using Student’s *t*-test for qualitative variables. The correlation between quality of life and coping strategies was determined using Spearman’s correlation test. To examine the predictive value of QOL for learning disability, a binary logistic regression model was developed, in which children with and without learning disability served as dichotomous variables. QOL and demographic variables that were observed to be significantly different between the two groups were considered independent variables. Hosmer-Lemeshow and R^2^ were computed to assess the model’s goodness of fit. Multiple regression models were used to determine the impact of various coping strategies on quality of life. Standardized beta values were used to interpret the findings of the regression analysis at the 95% confidence interval. Statistical significance was set at *p* < 0.05.

## 3. Results

Table 1 presents the demographic characteristics of the participants. This study invited 220 children living in different areas of the Alhasa region of Saudi Arabia. Of these 220 children, 97 of 115 (84.35%) with LD and 89 of 105 (91.30%) normative children fulfilled the study criteria. In total, 186 children with valid protocols were included in the final analyses. Compared to elementary and middle school students, high school participants had a significantly higher frequency of learning disability (χ^2^ = 5.44, *p* < 0.05).

In addition, it was also observed that the percentage of normal children was significantly higher in children belonging to joint families as compared to nuclear families (χ^2^ = 7.74, *p* < 0.01). Moreover, the findings revealed a significantly higher frequency of LD in patients with a poor socioeconomic status (<10,000 Saudi Riyal). No significant differences were found between these groups in terms of gender, age, area of residence, family occupation, or housing status.

Table 2 presents the mean scores and SDs of two groups of participants for the measures of QOL and coping strategies, along with the t-values. For the measure of QOL, results revealed significant differences between mean scores of two groups of participants for the measures of role functioning (*t* = −2.67, *p* < 0.01), bodily pain (*t* = −1.88, *p* < 0.05), general health (*t* = −3.23, *p* < 0.00), vitality (*t* = −4.51, *p* < 0.01), social functioning (*t* = −6.57, *p* < 0.01), role emotion (*t* = −1.87, *p* < 0.05) and mental health (*t* = −5.08, *p* < 0.01).

Mean scores clearly revealed that participants with LD have shown poor QOL in terms of role functioning (*M* = 63.40, *SD* = 28.88), bodily pain (*M* = 71.87, *SD* = 31.04), general health (*M* = 63.65, *SD* = 33.27), vitality (*M* = 55.67, *SD* = 30.30), social functioning (*M* = 38.76, *SD* = 27.81), role emotion (*M* = 61.34, *SD* = 43.60), and mental health (*M* = 51.44, *SD* = 23.58) in comparison to the normal children (Mean scores = 74.66, 79.49, 77.80, 73.59, 64.49, 72.69, 74.71; *SDs* = 28.53, 24.00, 25.40, 23.03, 25.36, 38.78, 37.78 respectively).

For different coping methods adopted by participants, significant differences were found in self-distraction (*t* = −4.60, *p* < 0.01), substance abuse (*t* = 4.47, *p* < 0.02), venting (*t* = −2.05, *p* < 0.05), positive reframing (*t* = −1.93, *p* < 0.05), religion (*t* = 1.93, *p* < 0.05), and self-blame (*t* = −2.48, *p* < 0.05). Participants with LD showed greater reliance on substance abuse (*M* = 3.23, *SD* = 1.87) and religious coping (*M* = 5.57, *SD* = 2.01) than normal children (mean scores = 2.27, and 5.20; *SDs* = 0.84, and 2.00, respectively). Normative children reported greater use of self-distraction coping (*M* = 5.48, *SD* = 1.62), venting (*M* = 4.91, *SD* = 1.87), positive reframing (*M* = 5.22, *SD* = 1.91), and self-blame (*M* = 4.41, *SD* = 1.89), than children with learning disability (Mean scores = 4.37, 4.35, 4.65, 3.76; *SDs* = 1.66, 1.84, 2.06, 1.68, respectively). However, the difference between the mean scores of these groups was not significant for active coping, denial, use of information support, behavioral disengagement, planning, humor, and acceptance.

The correlation between physical component summary and mental component summary aspects of QOL with different coping strategies was determined using Spearman’s correlation coefficient (Table 3). The results clearly revealed a fair to moderately strong correlation between physical component summary and all approaches to coping strategies except religious coping. Of all the approached of coping methods, we observed a weak correlation between denial (r = −0.17, *p* < 0.05), substance abuse (r = −0.15, *p* < 0.05), and behavioral disengagement (r = −0.18, *p* < 0.05), with a mental component summary aspect of QOL.

In addition to QOL and coping strategies, sociodemographic characteristics such as grade, family status, and monthly income were found to be significantly different between children with learning disability and healthy children. These confounding variables were incorporated as independent variables in a binary logistic regression model along with quality of life dimensions. Hosmer-Lemeshow statistics showed that there was no indication of a poor fit (*p* = 0.71). Table 4 presents the predictive value of each variable. The results of logistic regression analysis indicated that grade (OR = 3.79; *p* = 0.01) was significantly related to LD. The analysis revealed that LD was 3.79 times more likely in children studying in elementary classes. However, there was no significant association between LD and family status or monthly income.

In this study, we hypothesized that QOL can predict LD. The analysis showed that social health and mental health were significantly related to learning disability, even after regulating important confounders such as grade, family status, and monthly income.

As shown in Table 5, in the multiple regression model, the physical component summary score was significantly linked with denial (*β* = −0.33, CI = −6.87–−2.19, *p* < 0.01), and substance abuse (*β* = −0.14, CI = −4.96–0.40, *p* < 0.05). In the case of mental component summary, it was observed that active coping (*β* = −0.30, CI = −4.50–0.76, *p* < 0.01), behavioral disengagement (*β* = −0.20, CI = −4.48–0.30, *p* < 0.05), and humor coping strategy (*β* = 0.22, CI = 0.06–4.55, *p* < 0.05) had a significant impact on the SF-12 scores of children with LD. The coping strategies that had a significant impact on the overall QOL of children with LD were active coping, denial, and substance abuse.

## 4. Discussion

This study was conducted to investigate the differences in QOL and coping strategies between children with LD and healthy children. To the best of our knowledge, this is the first study conducted in Saudi Arabia to investigate the variations in coping mechanisms and QOL between children who have LD and those who do not. The findings of our study revealed significant differences between the two groups in role functioning, bodily pain, general health, vitality, social functioning, role emotion, and mental health dimensions of the SF-12. As expected, QOL using the SF-12 was poor in children with LD compared to healthy children, which seems to be mostly consistent with previous findings in healthy participants [35,36,37,38]. Children with LD often face challenges that affect their QOL. One reason for this could be LD can impact a child’s academic performance, leading to feelings of frustration, low self-esteem, and social stigmatization [39,40]. This can result in reduced opportunities for social interaction and participation in extracurricular activities, which are important for overall well-being. Furthermore, societal attitudes and misconceptions about learning disabilities can also play a role in affecting the quality of life of these children [41]. Stigma and discrimination can lead to feelings of isolation and inadequacy, affecting their mental health and overall happiness [42].

Coping strategies refer to the cognitive and behavioral actions taken to handle specific internal or external stressors perceived as challenging [43,44]. These strategies are typically categorized into groups such as adaptive and maladaptive, problem-focused or emotion-focused, and avoidant or approach-based coping [45]. However, there is limited research on the coping strategies used by children with learning disabilities, especially in Saudi Arabia. The current study identified six key coping strategies—self-distraction, substance use, venting, positive reframing, religion, and self-blame—that are significant in managing psychological distress. Prior research has shown that common coping methods for psychological distress among people with disabilities include seeking social support, problem-solving, physical activity, avoidance, engaging with social media, watching movies, and fostering relationships [46,47].

The analysis in this study revealed a significant difference in mean coping strategy scores between the two groups of children. Those with learning disabilities reported a higher reliance on substance use and religious coping strategies. A significant point here is that Saudi Arabia has relatively low rates of drug and alcohol usage. Although cultural attitudes may influence how these items are understood, subsequent conversations with participants indicated that some of the teenagers and their parents/caretakers in our study interpreted the Brief-COPE items on substance use in relation to prescribed and over-the-counter medications rather than illegal substances. It should also be noted that all children below 13 years of age scored zero for both substance abuse items. These findings partially align with previous research [48], which found that children with physical disabilities tend to use emotion-focused coping and avoidant coping strategies. Most research exploring the link between learning disabilities and substance use has focused on environmental factors rather than direct physiological connections. Currently, no substantial theory suggests a neurological or chemical mechanism directly linking learning disabilities to substance use. However, various studies have identified risk factors for substance use among children with learning disabilities, such as low self-esteem, academic difficulties, loneliness, depression, and the need for social acceptance [49,50,51,52,53,54,55]. Therefore, learning disabilities might indirectly lead to substance abuse by fostering behaviors that increase the likelihood of drug use among children. Moreover, religious coping involves seeking solace, guidance, or meaning through religious or spiritual beliefs and practices. Individuals with a learning disability may gravitate towards religion as a source of comfort or structure in their lives. Religious beliefs and practices can help people cope with difficult situations such as physical illnesses [56,57]. Results of our analysis also showed that healthy children rely more on self-distraction, venting, positive reframing, and self-blame coping to deal with their normal stress. Self-distraction coping involves diverting one’s attention from stressors or difficulties. Neurotypical children may employ this coping strategy more readily than children with learning disability because of differences in cognitive processing and social interaction. For example, normal children may engage in activities such as watching TV, playing video games, or spending time with friends to distract themselves from the stressors. Although there are no studies to which we can directly compare our findings, these findings are not compatible with other research that has revealed that optimism is a widely used method in children when they face a problem [58]. Venting refers to the act of expressing or releasing one’s feelings, frustrations, or emotions in a more intense or unrestricted manner. Venting emotions can provide a sense of release and relief, helping individuals to cope with stress or overwhelming feelings among children. Positive reframing involves a negative or challenging situation in a more positive way. Previous studies have noted that positive reframing was found to play a protective role against perceived stress and can be conceived as adaptive coping [59,60,61,62]. Self-blame involves attributing personal responsibility for a negative event or situation to oneself [63]. Previous research has indicated that children with high levels of self-blame are more likely to experience internalizing problems, whereas those with lower levels of self-blame do not show the same increase in such issues [64]. Children may indeed use self-blame as a coping mechanism in response to challenging situations. For example, children may feel overwhelmed by circumstances they cannot fully understand or control. By blaming themselves, they regain a sense of control over the situation, believing they can prevent similar occurrences in the future. Furthermore, children may use self-blame to protect relationships with caregivers or peers. They might believe that taking on the fault themselves can prevent conflict or avoid disappointing others.

This study emphasizes the role of coping strategies in improving the quality of life of children with learning disability by analyzing the relationship of each coping strategy. The discussion of theoretical underpinnings and empirical data clarifies the mechanism behind the observed correlation and function of coping strategies in the context of children’s disabilities. To enhance the quality of life for kids with learning disabilities, it also showed an association between adaptive and maladaptive coping mechanisms. The literature often distinguishes between adaptive and maladaptive coping mechanisms and shows that individuals may choose different strategies based on their specific circumstances, including the intensity of the stressor and individual characteristics, such as learning disabilities. Previous studies have demonstrated that individuals facing high-severity stressors are more likely to use problem-focused coping strategies, such as seeking solutions, while those facing lower-severity stress may rely more on emotion-focused strategies, like avoidance or denial. For instance, Folkman and Moskowitz (2004) highlight the importance of context and suggest that individuals assess the relevance and impact of stress before selecting their coping strategies [44]. Other research indicates that individuals with learning disabilities may experience unique challenges that can influence their coping strategies [65]. For example, they might face chronic academic stressors that require different coping mechanisms compared to their peers without learning disabilities.

Additionally, we assessed the relationship between demographic factors and QOL of children with LD. However, when sociodemographic characteristics were controlled for, and many differences between groups disappeared, it suggested that the variances in coping strategies might be more closely related to sociodemographic factors—such as age, gender, socioeconomic status, or cultural background—rather than the presence of learning disabilities themselves. Moreover, coping strategies employed by individuals can be significantly affected by their sociodemographic backgrounds. Factors like family income, education level, and social support can shape how individuals respond to stress, which means any observed differences in coping strategies may not be inherently linked to LD. If the differences in coping strategies are largely attributable to sociodemographic variables, interventions aimed at improving coping mechanisms might need to target these broader factors. Tailoring support and resources to address sociodemographic disparities could be beneficial for all individuals, including those with LD. The analysis indicated that grade was a significant predictor of QOL among children with LD. The results indicated that elementary school children were associated with LD. These results are similar to the previous studies, which examined that the prevalence of specific LD was higher in lower classes compared to higher classes [66]. Younger children are still developing foundational skills in reading, writing, and mathematics. Early identification of LD is crucial, and children in lower grades are more likely to be assessed and diagnosed as they are just beginning formal education. Moreover, the curriculum in lower grades emphasizes basic skills, making any learning challenges more apparent. As students progress to higher grades, the curriculum often becomes more specialized and may not highlight underlying LD as prominently.

The result of binary logistic regression analysis revealed that the social health and mental health of children were significantly associated with LD. The results suggested that social health was the best predictor of LD. The present findings lend support to the observations of previous research [67,68], which indicated that students with LD are less socially accepted [69,70], poor in developing close friendships [71,72], and are more victims of bullying [73,74]. Social health encompasses various aspects, such as social interactions, relationships, support systems, and emotional well-being. When a child or individual faces challenges in their social health, such as difficulties in forming relationships, social anxiety, or lack of support, it can impact their overall well-being and development. The results of our study also indicated that mental health was a significant predictor of LD. This result is in accordance with earlier research findings showing higher rates of anxiety and depression among individuals with learning disability [75,76,77,78]. LD and mental health issues can often co-occur in individuals. Learning disabilities refer to challenges in acquiring academic skills, while mental health issues encompass a wide range of conditions affecting one’s emotional well-being, behavior, and cognitive functions. Studies have indicated a higher prevalence of mental health issues [75], such as depression, anxiety, ADHD, and behavioral disorders, among individuals with learning disabilities compared to the general population. The presence of an LD can contribute to feelings of frustration, low self-esteem, and difficulties coping with academic demands, which can, in turn, increase the risk of developing mental health issues. Conversely, mental health issues can also impact a person’s ability to learn and succeed academically. Conditions like depression and anxiety can interfere with concentration, memory, and stress management, which are essential for effective learning. The interconnected nature of learning disabilities and mental health issues suggests a bidirectional relationship, where each condition can exacerbate the symptoms of the other. For example, a student with undiagnosed ADHD may struggle in school due to difficulty focusing, leading to feelings of inadequacy and eventually developing anxiety or depression. Therefore, through this logical analysis, we can conclude that the presence of mental health issues in individuals with learning disabilities is well-founded. Addressing both learning disabilities and mental health concerns through appropriate support, interventions, and accommodations is essential for promoting overall well-being and academic success in these individuals.

In the present study, remarkable findings were obtained. A physical component summary score of quality of life was significantly correlated with denial and substance abuse. These results are partially supported by previous studies [79], which indicated that families of children with learning disabilities adopted avoidant coping strategies. Denial coping involves avoiding or minimizing the reality of a situation. For people with LD, denying or downplaying the challenges they face may lead them to neglect their physical health needs. For example, someone in denial may not seek appropriate medical care or adhere to necessary treatment plans, which can negatively affect their physical well-being. Moreover, by using denial as a coping mechanism, individuals with learning disabilities may inadvertently compromise their physical health, ultimately affecting their overall quality of life. This could lead to decreased functional abilities, lower energy levels, and increased susceptibility to health complications. Furthermore, continuously employing denial as a coping strategy may have long-term consequences on physical health outcomes. Ignoring or denying the impact of a learning disability on one’s physical well-being can prevent individuals from taking proactive steps to address health issues early on, potentially leading to more severe health problems down the line. Previous studies have reported a link between poor quality of life and drug abuse [80,81]. Our results indicated a connection between poor QOL and drug abuse among individuals with learning disabilities. Individuals with learning disabilities may face challenges in social interactions, education, employment, and daily living activities, which can contribute to feelings of isolation, low self-esteem, and higher levels of stress. This can, in turn, make them more vulnerable to seeking solace or escape through substance abuse. Moreover, issues such as lack of access to appropriate support services, stigma, discrimination, and limited opportunities for personal growth may further exacerbate the risk of drug abuse among this population. It is crucial for healthcare providers and support systems to address the unique needs of individuals with learning disabilities, to provide tailored interventions and support to enhance their quality of life and reduce the likelihood of engaging in substance abuse.

Interestingly, a mental component summary score of SF-12 was associated with active coping, behavioral disengagement, and humor. Our results are partially supported by previous studies [82], which demonstrated that patients with multiple sclerosis used acceptance and active coping strategies. Considering the research findings, it is important to emphasize that combining problem- and emotion-focused coping strategies is regarded as an adaptive method of handling stressful circumstances [83]. The mental component summary score of the SF-12 reflects an individual’s mental health status, including aspects such as emotional well-being, psychological distress, and social functioning. The associations between the mental component summary score of the SF-12 and the coping strategies of active coping, behavioral disengagement, and humor illustrate how mental health significantly influences an individual’s approach to challenges. Those with higher mental component summary scores are empowered to engage actively with their challenges, while those with lower scores may experience stress and disengagement. Humor acts as a positive coping mechanism for those with better mental health, suggesting a holistic relationship between mental health status and adaptive coping strategies in individuals with learning disabilities. Thus, fostering better mental health should be a priority in supporting individuals with learning disabilities, as it directly impacts their coping mechanisms and overall well-being.

While this study demonstrated an association between the QOL and coping strategies among children with and without learning disabilities, it has some limitations. Notably, the small sample size limits the representativeness of the results and their generalizability to the broader population. The second limitation of this study was the selection bias in the participant recruitment. Recruiting children from clinics, special education schools, and support centers, the sample may not fully represent the broader population of children with learning disability. These settings may attract individuals with specific characteristics or access to resources, thus potentially skewing our findings. The third limitation was only to focus on children aged 6–18 years, which limits the generalizability to older or younger age groups. Relying solely on parent/caregiver reports for demographic and clinical variables introduces the possibility of response bias or inaccuracies, which was another limitation of this study. The study’s cross-sectional design also limits the ability to establish causal relationships between coping strategies and QOL. Moreover, we attempted to match participants based on demographic criteria such as age, location, and ethnicity. However, we were unable to match siblings or family members, which may impact the results added to limitations. The tool used for data collection was designed mainly for 18 years and above, which might have some influence on the results. Longitudinal studies can provide deeper insights into these associations over time. Finally, while the study aimed to adhere to ethical guidelines, variations in the interpretation or implementation of these guidelines across different institutions may introduce inconsistencies in ethical processes.

## 5. Conclusions

This study explored QOL and coping strategies of children with and without LD. The results of our study revealed significant differences between the two groups in role functioning, bodily pain, general health, vitality, social functioning, role emotion, and mental health dimensions of the SF-12. QOL was poor in children with LD compared to healthy children. The findings of the present study revealed that self-distraction, substance abuse, venting, positive reframing, religion, and self-blame have been identified as important in dealing with psychological distress. Participants with LD reported a greater use of substance abuse and religious coping. A further exploration of these variables may be needed. We recommend future researchers examine the cultural validity and interpretation of these items with caution, particularly among adolescents in Muslim countries. Healthy children rely more on self-distraction venting, positive reframing, and self-blame coping to deal with their normal stress. Additionally, we assessed the relationship between demographic factors and QOL of children with learning disability. The results indicated that elementary school children were associated with LD. The results of binary logistic regression analysis revealed that social health and mental health were the best predictors of LD. The Ministry of Health, Ministry of Education, and other relevant organizations may find this study valuable for enhancing coping strategies aimed at reducing psychological distress in individuals with learning disabilities. Future research should explore coping strategies and related factors in greater depth by employing diverse methods, including various study designs and variables, and by examining different regions of the country to gain a comprehensive understanding of quality of life and coping mechanisms.

## Figures and Tables

**Table 1:** Demographic Characteristics of Children with and without Learning Disability.

Variables	LD Children n = 97 n (%)	Normal Children n = 89 n (%)	Chi Square Test	*p* Value
Gender			1.24	0.27
Male	58 (59.79%)	46 (51.69%)		
Female	39 (40.21%)	43 (48.31%)		
Age			2.71	0.97
6–12 years	45 (46.39%)	42 (47.19%)		
13–18 years	52 (53.61%)	47 (52.81%)		
Grade			5.44	0.05 *
Elementary	30 (30.93%)	13 (14.61%)		
Middle	26 (26.80%)	41 (46.07%)		
High school	41 (42.27%)	35 (39.32%)		
Family status			7.74	0.01 **
Joint	31 (31.96%)	86 (96.63%)		
Nuclear	66 (68.04%)	3 (3.37%)		
Area of residence			1.35	0.25
Urban	90 (92.78%)	85 (95.50%)		
Rural	7 (7.22%)	4 (4.50%)		
Monthly income			12.90	0.01 **
<10,000 Saudi Riyal	60 (61.86%)	41 (46.06%)		
10,001–15,000 Saudi Riyal	29 (29.90%)	23 (25.85%)		
>15,001 Saudi Riyal	8 (8.25%)	25 (28.09%		
Family occupation			1.81	0.72
Government employees	50 (51.55%)	47 (52.81%)		
Private employee	24 (24.74%)	17 (19.10%)		
Business	23 (23.71%)	25 (28.09%)		
Housing status			2.78	0.09
Own	66 (68.04%)	54 (60.67%)		
Rented	31 (31.96%)	35 (39.33%)		

Note: * *p* < 0.05, ** *p* < 0.01.

**Table 2:** Quality of Life and Coping Strategies among Patients with Learning Disability and Healthy Children.

	LD Children		Normal Children			
Quality of Life	*M*	*SD*	*M*	*SD*	*t*-Value	*p* Value
Role Functioning	63.40	28.88	74.66	28.53	−2.67	0.01 **
Role Physical	55.41	37.01	58.42	21.30	0.67	0.50
Bodily Pain	71.87	31.04	79.49	24.00	−1.88	0.05 *
General Health	63.65	33.27	77.80	25.40	−3.23	0.01 **
Vitality	55.67	30.30	73.59	23.03	−4.51	0.01 **
Social Functioning	38.76	27.81	64.49	25.36	−6.57	0.01 **
Role Emotion	61.34	43.60	72.69	38.78	−1.87	0.05 *
Mental Health	51.44	23.58	74.71	37.78	−5.08	0.01 **
**Coping Strategies**						
Self-Distraction	4.37	1.66	5.48	1.62	−4.60	0.01 **
Active Coping	4.57	1.94	4.91	1.96	−1.16	0.24
Denial	3.64	1.74	4.03	1.84	−1.46	0.15
Substance Abuse	3.23	1.87	2.27	0.84	4.47	0.01 **
Emotional Support	4.38	1.82	4.51	1.75	−0.51	0.61
Use of Information Support	4.46	1.80	4.85	1.77	−1.48	0.14
Behavioral Disengagement	3.80	1.79	3.96	1.52	−0.66	0.51
Venting	4.35	1.84	4.91	1.87	−2.05	0.04 *
Positive reframing	4.65	2.06	5.22	1.91	−1.93	0.05 *
Planning	4.77	2.03	5.07	1.78	−1.08	0.28
Humor	3.73	1.73	3.71	1.64	0.05	0.96
Acceptance	4.81	1.96	5.32	1.97	−1.77	0.08
Religion	5.77	2.01	5.20	2.00	1.93	0.05 *
Self-blame	3.76	1.68	4.41	1.89	−2.48	0.01 **

Note: ** *p* < 0.01; * *p* < 0.05.

**Table 3:** Correlation between Quality of life and various Coping Strategies.

	1	2	3	4	5	6	7	8	9	10	11	12	13	14	15	16
1. PCS	01:00															
2. MCS	0.13	01:00														
3. SD	−0.20 **	0.00	01:00													
4. AC	−0.18 *	−0.11	0.37 **	01:00												
5. DN	−0.45 **	−0.17 *	0.27 **	0.25 **	01:00											
6. SA	−0.27 **	−15 *	0.16 *	0.06	0.32 **	01:00										
7. ES	−0.27 **	0.02	0.44 **	0.57 **	0.31 **	0.26 **	01:00									
8. US	−0.23 **	−0.01	0.35 **	0.55 **	0.32 **	0.19 **	0.62 **	01:00								
9. BD	−0.31 **	−0.18 *	0.31 **	0.32 **	0.53 **	0.55 **	0.36 **	0.43 **	01:00							
10. VT	−0.33 **	−0.01	0.37 **	0.52 **	0.47 **	0.23 **	0.60 **	0.60 **	0.49 **	01:00						
11. PR	−0.21 **	−0.05	0.41 **	0.63 **	0.31 **	0.09	0.50 **	0.48 **	0.38 **	0.64 **	01:00					
12. PL	−0.23 **	−0.01	0.51 **	0.59 **	0.36 **	0.20 **	0.54 **	0.55 **	0.37 **	0.60 **	0.65 **	01:00				
13. HU	−0.25 **	−0.05	0.30 **	0.41 **	0.43 **	0.46 **	0.35 **	0.36 **	0.69 **	0.50 **	0.49 **	0.38 **	01:00			
14. AT	−0.16 *	0.06	0.43 **	0.57 **	0.23 **	0.13	0.54 **	0.46 **	0.27 **	0.49 **	0.62 **	0.64 **	0.34 **	01:00		
15. RL	0.00	0.03	0.16 *	0.31 **	0.05	0.05	0.26 **	0.22 **	0.08	0.25 **	0.26 **	0.30 **	0.11	0.31 **	01:00	
16. SB	−0.28 **	−0.08	0.34 **	0.25 **	0.53 **	0.28 **	0.20 **	0.36 **	0.46 **	0.48 **	0.37 **	0.45 **	0.47 **	0.33 **	0.08	10.00

Note: PCS = Physical Component Summary; MCS = Mental Component Summary; SD = Self-Distraction; AC = Active Coping; DN = Denial; SA = Substance Abuse; ES = Emotional Support; US = Use of Information Support; BD = Behavioral Disengagement; VT = Venting; PR = Positive Reframing; PL = Planning; HU = Humor; AT = Acceptance; RL = Religion; SB = Self-blame. ** *p* < 0.01; * *p* < 0.05.

**Table 4:** Result of Binary Logistic Regression Model for Quality of Life of Children with Learning Disability.

Variables	OR	95% CI	*p* Value
Grade (ref: High school)			
Elementary	3.79	1.44–9.91	0.01 **
Middle	1.74	0.57–5.27	0.33
Family status (ref: Nuclear)			
Joint	0.43	0.15–1.19	0.11
Monthly income (ref: >15,001 Saudi Riyal)			
<10,000 Saudi Riyal	0.08	0.06–0.95	0.15
10,001–15,000 Saudi Riyal	0.10	0.05–0.89	0.19
Quality of life			
Social health	1.04	1.02–1.06	0.01 **
Mental health	1.03	1.00–1.04	0.02 *

Note: * *p* < 0.05, ** *p* < 0.01 Abbreviations: OR, odd ratio.

**Table 5:** Impact of Coping Strategies on Quality of Life of children with Learning Disability after controlling the Demographic characteristics (Grade, Family status, and Monthly income).

Variables	Unstandardized Coefficient		Standardized Coefficient	95% CI	*p* Value
	B	SE	Beta		
Regression Model for Physical Component Summary					
Self-Distraction	−0.62	1.14	−0.04	−2.87–1.63	0.58
Active Coping	−0.43	1.24	−0.03	−2.87–2.03	0.73
Denial	−4.53	1.18	−0.33	−6.87–−2.19	0.01 **
Substance Abuse	−2.27	1.35	−0.14	−4.96–0.40	0.05 *
Emotional Support	−1.27	1.42	−0.93	−4.07–1.53	0.37
Use of Information Support	0.10	1.31	0.01	−2.50–2.69	0.94
Behavioral Disengagement	0.34	1.58	0.02	−2.78–3.47	0.83
Venting	−1.67	1.41	−0.13	−4.45–1.12	0.24
Positive reframing	0.22	1.32	−0.02	−2.82–2.38	0.87
Planning	0.37	1.37	0.03	−2.35–3.08	0.79
Humor	0.74	1.49	0.05	−2.19–3.68	0.62
Acceptance	0.50	1.20	0.41	−1.87–2.87	0.68
Religion	0.90	0.86	0.07	−0.80–2.60	0.30
Self-blame	−0.28	1.23	−0.02	−2.71–2.15	0.82
Regression Model for Mental Component Summary					
Self-Distraction	0.25	0.87	0.03	−1.46–1.97	0.77
Active Coping	−2.63	0.95	−0.30	−4.50–0.76	0.01 **
Denial	−1.21	0.91	−0.13	−3.00–0.57	0.18
Substance Abuse	−1.81	1.03	−0.16	3.85–0.22	0.08
Emotional Support	0.81	1.08	0.08	−1.32–2.95	0.46
Use of Information Support	0.73	1.01	0.07	−1.25–2.71	0.47
Behavioral Disengagement	−2.09	1.21	−0.20	−4.48–0.30	0.05 *
Venting	0.90	1.08	0.98	−1.22–3.03	0.40
Positive reframing	−1.16	1.01	−0.13	−3.15–0.83	0.25
Planning	0.52	1.05	0.06	−1.55–2.59	0.62
Humor	2.30	1.14	0.22	0.06–4.55	0.04 *
Acceptance	1.54	0.92	0.17	−0.27–3.35	0.09
Religion	0.30	0.66	0.03	0.99–1.60	0.65
Self-blame	−0.44	0.94	−0.04	−2.30–1.42	0.64
Regression Model for Overall Quality of Life					
Self-Distraction	−0.36	1.47	−0.20	−3.28–2.54	0.80
Active Coping	−3.06	1.60	−0.19	−6.23–0.12	0.05 *
Denial	−5.75	1.53	−0.33	−8.78–−2.72	0.01 **
Substance Abuse	−4.09	1.75	0.20	−7.55–−063	0.02 *
Emotional Support	−0.46	1.84	−0.03	−4.09–3.17	0.80
Use of Information Support	0.83	1.70	0.05	−2.53–4.19	0.63
Behavioral Disengagement	−1.74	2.05	−0.09	−5.79–2.30	0.39
Venting	−0.76	1.83	−0.04	−4.37–2.85	0.68
Positive reframing	−1.38	1.71	−0.08	−4.76–1.99	0.42
Planning	0.88	1.78	0.05	−2.63–4.40	0.62
Humor	3.05	1.93	0.16	−0.75–6.86	0.12
Acceptance	2.04	1.56	0.13	−1.04–5.12	0.19
Religion	1.20	1.12	0.07	−1.00–3.40	0.28
Self-blame	−0.72	1.60	−0.04	−3.87–2.43	0.65

Note: * *p* < 0.05, ** *p* < 0.01.

## Data Availability

The Data that support our findings can be found by directly asking the corresponding author.
